# ARE PAIN APPS USABLE? MHEALTH LITERACY FRAMEWORK

**DOI:** 10.1093/geroni/igac059.2219

**Published:** 2022-12-20

**Authors:** Mary Milidonis, Carolyn Menges

**Affiliations:** Cleveland State University, HUDSON, Ohio, United States; Cleveland State University, Cleveland, Ohio, United States

## Abstract

Older adults are at risk for low health literacy and not adhering to self-care. Health professionals are shifting their practices to emphasize wellbeing through health promotion. Mobile pain applications may be a tool to improve health communication and individualize care. Mobile health apps do not always consider the needs of the older adult who may be less comfortable and confident with technology. The purpose of this qualitative review is to explore mobile pain applications for adults with a health literacy framework, and identify facilitators and barriers to usability. Databases that reviewed apps included Google, Google Scholar, ScienceDirect, PubMed, CINAHL plus full text, APTA EBP database, MEDline, and SportDiscus. Fifty-three apps found. Excluded criteria: if there was a cost, targeted for children, and without pain diary. Six apps identified to be specifically relevant to pain intensity, location quality, and impact on life. Mobile Application Rating System (MARS) tool assessed engagement, functionality, aesthetics, information quality, and subjective quality. All apps were engaging, allowed for tracking symptoms and life impact over time. Some apps were difficult to navigate, did not offer education support. Two of the apps individualized pain. Two apps are on only accessible on one app platform. One app allowed for feedback about the app design. Pain mobile health applications can improve tracking, managing and understanding pain for improved mobility and social engagement for older adults. The use of health literacy frameworks with mobile health applications may increase accessibility to care.

